# A Comparative Analysis of Breast Cancer Detection and Diagnosis Using Data Visualization and Machine Learning Applications

**DOI:** 10.3390/healthcare8020111

**Published:** 2020-04-26

**Authors:** Muhammet Fatih Ak

**Affiliations:** Industrial Engineering Department, Antalya Bilim University, 07190 Antalya, Turkey; fatih.ak@antalya.edu.tr; Tel.: +90-242-245-0000

**Keywords:** breast cancer, data visualization, early diagnosis, machine learning, risk assessment

## Abstract

In the developing world, cancer death is one of the major problems for humankind. Even though there are many ways to prevent it before happening, some cancer types still do not have any treatment. One of the most common cancer types is breast cancer, and early diagnosis is the most important thing in its treatment. Accurate diagnosis is one of the most important processes in breast cancer treatment. In the literature, there are many studies about predicting the type of breast tumors. In this research paper, data about breast cancer tumors from Dr. William H. Walberg of the University of Wisconsin Hospital were used for making predictions on breast tumor types. Data visualization and machine learning techniques including logistic regression, k-nearest neighbors, support vector machine, naïve Bayes, decision tree, random forest, and rotation forest were applied to this dataset. R, Minitab, and Python were chosen to be applied to these machine learning techniques and visualization. The paper aimed to make a comparative analysis using data visualization and machine learning applications for breast cancer detection and diagnosis. Diagnostic performances of applications were comparable for detecting breast cancers. Data visualization and machine learning techniques can provide significant benefits and impact cancer detection in the decision-making process. In this paper, different machine learning and data mining techniques for the detection of breast cancer were proposed. Results obtained with the logistic regression model with all features included showed the highest classification accuracy (98.1%), and the proposed approach revealed the enhancement in accuracy performances. These results indicated the potential to open new opportunities in the detection of breast cancer.

## 1. Introduction

Data science has become one of the most popular research areas of interest in the world. Many datasets can be useful in different situations such as marketing, transportation, social media, and healthcare [1]. However, only a few of them have been interpreted by data science researchers, and they believe that these datasets can be useful for predictions. Nowadays, many of the marketers have started to analyze their datasets because of the big information they have on hand, and they want to turn these data into meaningful information for future predictions. By doing that, marketers can apply some new tactics or change their goal [2].

Data mining and machine learning techniques are straightforward and effective ways to understand and predict future data. Dealing with large data manually is almost impossible [3]. Therefore, data visualization is a very important step to have a general idea about given data. Data analysis techniques are popular in many companies and have an impact on different study areas. For instance, Facebook’s News Feed uses machine learning by following user patterns [4]. Another study has been made about optimizing energy consumption in large-scale buildings [5]. Customer relationship management systems are also using machine learning techniques [6]. In addition to all these different studies, machine learning studies in healthcare are very popular [7]. Data mining techniques and clustering methods are used for different types of diseases to make data understandable and teach the computer to predict current data.

Cancer death is one of the major issues for the healthcare environment. It is one of the most significant reasons for women’s death [8]. Breast cancer is the most common type of cancer in women with denser breast tissue due to its physiological features. The detection of this disease in the early stages can help to avoid the rising number of deaths [8]. According to the Globocan 2018 data, one of every four cancer cases diagnosed in women worldwide is breast cancer, and it ranks fifth among the causes of death worldwide [9]. According to the same data, the incidence of age-related breast cancer worldwide in 2018 was 23.7 per 100,000, whereas the mortality rate due to breast cancer was reported as 6.8 per 100,000 [9]. Despite the increase in the number of medical studies and technological developments that contribute to the treatment of cancer, there are still some problems in the diagnosis of cancer. After lung cancer, breast cancer is the major cause of women’s death [9]. Breast cancer originates from breast tissue, most commonly from the inner lining of milk ducts or the lobules that supply the ducts with milk [10,11]. A mutation or modification of DNA or RNA could force normal cells to transform into cancer cells, and these mutations could occur due to an increase in entropy or nuclear radiation, chemicals in the air, bacteria, fungi, electromagnetic radiation viruses, parasites, heat, water, food, mechanical cell-level injury, free radicals, evolution, and aging of DNA and RNA [12]. It is important to make an accurate diagnosis of tumors. Most tumors are the result of benign (non-cancerous) changes within the breast, but if a malignant tumor is diagnosed as benign it will cause serious problems. Early detection of breast cancer and getting modern cancer treatment are the most important strategies to prevent deaths from breast cancer. It is easy to treat early, small, and non-spreading breast cancer successfully. The most reliable way to find breast cancer early is by having regular screening tests.

Age, family history, genetics, race, ethnicity, being overweight, drinking alcohol, and lack of exercise are risk factors associated with breast cancer [13]. Healthcare is an open-ended environment with very rich information, yet very poor knowledge. There is a huge amount of data in healthcare systems, and it is important to discover and build relationships with hidden data. The main causes of death were classified into five broad groups according to the International Classification of Diseases (ICD), and breast cancer was included in two groups [14]. A report from McKinsey states that the volume of data is growing at a rate of 50% per year [15]. Currently, data science has officially become a very significant field even though the term data science was first coined in the early 1990s. A study defined the term data science as implying focus around data and, by extension, statistics, which is a systematic study about the organization, properties, and analysis of data and their role in inference [16]. In previous data mining research about healthcare, some methods were applied to different types of diseases and genes, and the methods including analytical, collecting, sharing, and compressing methods were applied on healthcare datasets [17]. Even though multiple disciplines can be applied to data science, machine learning methods are mostly applied to healthcare datasets. Machine learning is a data analysis technique that teaches a computer what comes as an output with different algorithms. Decision tree, k-means clustering, and neural networks are the most common algorithms for machine learning applications [18]. While there is no better way to diagnose breast cancer, early diagnosis can be accepted as the first step of treatment and risk assessment to minimize factors. It allows a person to control risk factors, although some breast cancer risk factors cannot be changed. 

In this study, public data about breast cancer tumors from Dr. William H. Walberg of the University of Wisconsin Hospital were taken and used for data visualization, classification, and machine learning algorithms, which included logistic regression, k-nearest neighbors, support vector machine, and decision tree [19]. Public data included samples taken from patients with solid breast masses and a user-friendly usage of graphical programs called City. This study aimed to establish an adequate model by revealing the predictive factors of early-stage breast cancer patients from a wider perspective and compare the strength of the model with accuracy measures. 

The organization of the remaining sections of the current study is as follows. A literature review that contains recent related studies on breast cancer detection and diagnosis is in Section 2. In Section 3 and Section 4, methods and application of the proposed method to a dataset are given. The results, discussion, and comparative analysis are demonstrated in the last section.

## 2. Literature Review 

The healthcare environment is one of the most accurate fields for data science applications due to the amount of data that it contains and the suitability of data type. The flow of data in hospitals is a continuous process and includes numerical values in general. Healthcare is an open system for improvements with studies about data mining and machine learning techniques. Dhar claims that expertise on a computer would give you significant results and the possibility to predict the future with given data [14]. There are many studies done on breast cancer datasets, and most of them have sufficient classification accuracy [20,21]. 

Aruna et al. [22] used naïve Bayes, support vector machine, and decision trees to classify a Wisconsin breast cancer dataset and got the best result by using support vector machine (SVM) with an accuracy score of 96.99%. Chaurasia et al. [23] compared the performance of supervised learning classifiers by using a Wisconsin breast cancer dataset and naïve Bayes, SVM, neural networks, decision tree methods applied. According to the study results, SVM gave the most accurate result with a score of 96.84%. Asri et al. [24] also used the same data and made a performance comparison among machine learning algorithms: SVM, decision tree (c4.5), naïve Bayes, and k-nearest neighbors. The study aimed to classify data in terms of efficiency and effectiveness by comparing the accuracy, precision, sensitivity, and specificity of each algorithm. The experimental result showed that SVM had the best score with an accuracy of 97.13%. Delen et al. [25] studied the prediction of breast cancer data with 202,932 patient records. The dataset was divided into two different groups as survived (93,273) and not survived (109,659), then naïve Bayes, neural network, and c4.5 decision tree algorithms were applied. The achieved results showed that the c4.5 decision tree had better performance than the other techniques.

Ou et al. [26] made a comparison between naïve Bayes, decision tree, and random tree to get the best results for classifying the Diabetic disease dataset. From the findings of this study, naïve Bayes was decided as the best classifier with a score of 76.3%. Srinivas et al. [27] studied one dependency augmented naïve Bayes classifier and naïve creedal classifier to make predictions on heart attacks by using medical profiles such as age, sex, blood pressure, and blood sugar. The study result indicated that naïve Bayes observed better results. Bernal et al. [28] used clinical data on medical intensive care units. Machine learning techniques such as logistic regression, neural networks, decision tree, and k-nearest neighbors were applied to predict the decrease of patients inside the hospital over 24 hours. The highest accuracy scores were obtained with logistic regression and k-nearest neighbor (KNN)-5 technique among training data. Bernal [28] pointed out that it is necessary to decide parameters rather than the algorithm to get better accuracy results.

Wang et al. [29] studied to find the best way for breast cancer predictions by using data mining methods on several records. They applied support vector machine (SVM), artificial neural network (ANN), naïve Bayes classifier, and AdaBoost Tree. Reducing the feature space was discussed, then Principle Component Analysis (PCA) was applied with the aim of reduction. In the evaluation part of the performance of the models, they used two datasets that were the Wisconsin Breast Cancer Database (1991) and Wisconsin Diagnostic Breast Cancer (1995) [18,30]. They provided a detailed evaluation of the models and test errors. 

Williams et al. [31] made studies about risk prediction on breast cancer by using data mining classification techniques. Breast cancer is the most common cancer type for women throughout Nigeria. There are limited services to predict breast cancer before it is too late to aid. So, they needed to obtain an efficient way to predict breast cancer. Two data mining techniques used in their study were naïve Bayes and the J48 decision trees. 

Nithya et al. [32] believe that the main problem of breast cancer is about classifying the breast tumor. Computer-Aided Diagnosis (CAD) has been used for the detection and characterization of breast cancer. Their main idea was improving breast cancer prediction by using data mining methods. Bagging, multiboot, random subspace to the classification performance of naïve Bayes, support vector machine-sequential minimal optimization (SVM-SMO), and multilayer perceptron were applied.

Oyewola et al. [33] made investigations on breast cancer biopsy predictions with a mammographic diagnosis. Logistic regression (LR), linear discriminant analysis (LDA), quadratic discriminant analysis (QDA), random forest (FR), and support vector machine (SVM) classifications were used in their study. 

Agarap [34] used SVM, LR, multilayer perceptron, KNN, softmax regression, SVM, and Gated Recurrent Unit (GRU) SVM techniques. The most reliable result was obtained from a multilayer perceptron with an accuracy score of 99.4%.

Westerdijk [35] studied several machine learning techniques for the prediction of breast cancer cells. She tested the performance of the models by looking at their accuracies, sensitivities, and specificities. Accuracy scores of LR, random forest, SVM, neural network, and ensemble models were compared. The prediction of breast cancer should be improved with the accuracy score.

Vard et al. [36] studied a robust method for predicting eight cancer types such as breast cancer, lung cancer, and ovarian cancer. In their research, firstly they used Particle Swarm Optimization to normalize datasets and statistical feature selection methods to separate features on a normalized dataset. They then applied decision tree, support vector machines, and a multilayer perceptron neural network for classifications. 

Kourou et al. [37] investigated the classification of cancer patients’ risk groups in two types as low and high. ANN, Bayesian networks (BNs), SVM, and decision tree (DT) techniques were used to present a model for cancer risks or patient outcomes. 

Pratiwi [38] considered breast cancer is the most common death reason for women. Machine learning techniques were preferred to diagnose breast cancer. Pratiwi used Java to develop intelligent breast cancer prediction, proving that all functionalities worked well and were done without significant delay.

Shukla et al. [39] studied a robust data analytical model that could be useful on breast cancer datasets. The model included the survivability of patients and tumors. They used the Surveillance, Epidemiology, and End Results (SEER) program to identify, and the Self-Organizing Map (SOM) and Density-Based Spatial Clustering of Applications with Noise (DBSCAN) for clustering. Table 1 summarizes recent related studies, their aim, and approach.

Clarification of data science [10,11,12,13,14,15,16,17,18,19,20,21,22] and prediction of breast cancer by using data mining techniques [23,24,25,26,27,28,29,30,31,32,33] are objectives of the recent studies as two main categories. Experimental results showed that the best score had an accuracy of 97.13% [19].

The main contributions of this paper are provided in the following:

- Establish an adequate model by revealing the predictive factors of early-stage breast cancer patients from a broader perspective and compare the robustness of the model by accuracy measures;
- A more comprehensive comparison and analysis using data visualization and machine learning applications for breast cancer detection and visibility to validate the model;
- Observe which features are most effective in predicting breast cancer and to understand general trends;
- A better prediction of breast cancer by using data mining methods.

## 3. Methods and Application

### 3.1. Logistic Regression 

**Figure 1:**
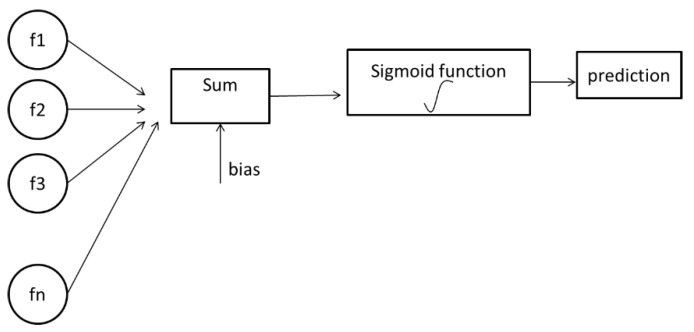
Visualization of logistic regression steps.

Logistic regression is a technique that firstly used for biological studies in the early twentieth century. It has become widespread for social studies too. Logistic regression is also one of the predictive analyses. Logistic regression is appropriate to use when there is one binary dependent variable and other independent variables. Linear and logistic regressions are different in terms of the dependent variable. Linear regression is a more appropriate technique for continuous variables. Figure 1 indicates the visualization of logistic regression steps.

Logistic regression has two phases: forward propagation and backward propagation. The first step of forward propagation is multiplying weights with features. Initially, since weights are unknown, random values can be assigned. A sigmoid function assigns a probability between 0 and 1. According to a threshold value, the prediction is performed. After prediction, the predictive value is compared with the observed values, and then a loss function is generated. The loss function indicates how far the predicted value is from the real value. If the loss function value is very high, then backward propagation is applied. The aim of backward propagation is updating weight values according to cost function by taking the derivative [40]. The sigmoid function is shown below:

(1) $$\sigma \left(z\right)=\frac{1}{1+e^{-z}}$$

### 3.2. K-Nearest Neighbor (KNN)

**Figure 2:**
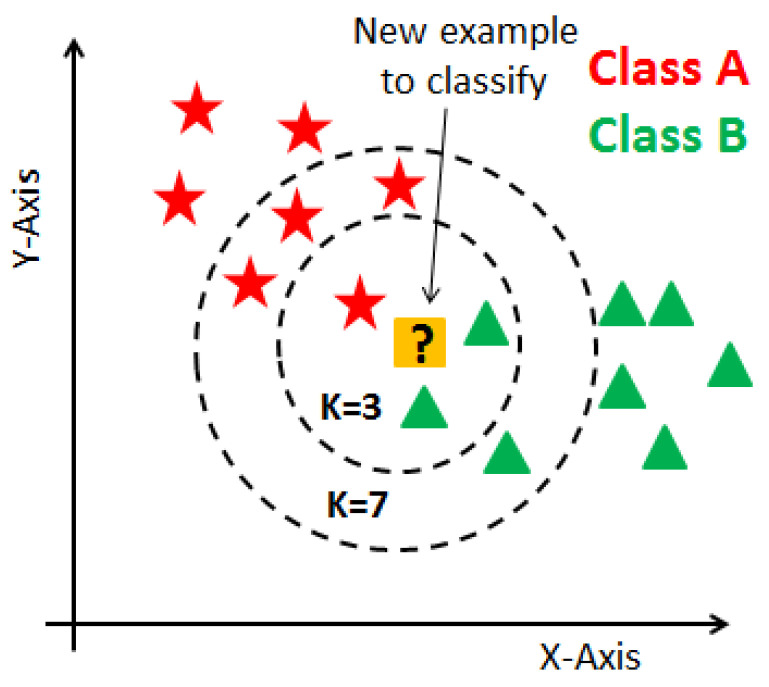
Representation of KNN algorithm [12].

KNN is a supervised learning technique that means the label of the data is identified before making predictions. Clustering and regression are two purposes to use it. K represents a numerical value for the nearest neighbors. KNN algorithm does not have a training phase. Predictions are made based on the Euclidean distance to k-nearest neighbors. This technique is applied to the prediction of breast cancer dataset since it already has labels such as malignant and benign. The label is classified according to the nearest neighbor to the class labels of its neighbors. A representation of the KNN algorithm is shown in Figure 2.

### 3.3. Support Vector Machine 

**Figure 3:**
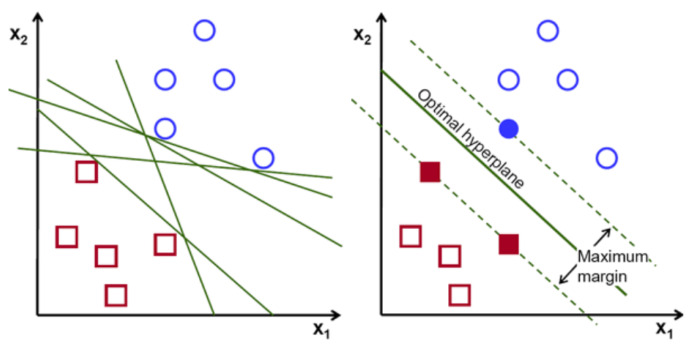
Support vector visualization [8].

Support vector machine is one of the most common machine learning techniques. The objective of the algorithm is to find a hyperplane in N-dimensions that classifies the data points. The major part of this algorithm is finding the plane that maximizes the margin. N dimension diversifies based on the feature numbers. Comparing two features could be done smoothly. However, if there are several features for classification, it is not always that straightforward. Maximizing the margin provides more accurate prediction results [41]. Figure 3 indicates the visualization of SVM.

SVM has a small tradeoff between large margin and accurate classification. If the exact classification without sacrificing any individual sample is applied, the margin could be very narrow, which could lead to a lower accuracy level. On the other hand, by maximizing the margin between classes to get a better accuracy, support vectors that are closest to the hyperplane could be considered with other class members.

### 3.4. Naïve Bayes

Naïve Bayes is a straightforward and also fast algorithm for classification. Its working process is based on Bayes theorem. It is represented below:

(2) $$P\left(X|Y\right)=\frac{P\left(Y|X\right)P\left(X\right)}{P\left(Y\right)}$$

The fundamentals of this algorithm assume that each variable contributes to the outcome independently and equally. In this case, each feature will not be dependent on each other and will affect the output with the same weight. Therefore, the naïve Bayes theorem does not apply to real-life problems, and it is possible to get low accuracies while using this algorithm. Gaussian Naïve Bayes is one kind of naïve Bayes application. It assumes that features follow a normal distribution. The possibility of features is considered to be Gaussian and has a conditional probability. Gaussian naïve Bayes theorem is given below:

(3) P(xi|y)=12πσ^2yexp(−(xi−μy)22σ2y)

### 3.5. Decision Tree

A decision tree (DT) is one of the most common supervised learning techniques. Regression and classification are two main goals to use it. It seeks to solve problems by drawing a tree figure. Features are known as decision nodes, and outputs are leaf nodes. Feature values are considered as categorical in the decision tree algorithm. At the very beginning of this algorithm, it is essential to choose the best attribute and place it at the top on tree figure and then split the tree. Gini index and information gain are two methods for the selection of features.

Randomness or uncertainty of feature x is defined as entropy and can be calculated as follows:

(4) $$H\left(x\right)=Ex\left[I\left(x\right)\right]=-\sum^{}p\left(x\right)logp\left(x\right)$$

Entropy values for each variable are calculated, and by subtracting these values from one, information values can be obtained. A higher information gain makes an attribute better and places it on top of the tree.

Gini index is a measure of how often a randomly chosen element would be incorrectly identified. Therefore, a lower Gini index value means better attributes. Gini index can be found with the given formula:

(5) $$G=\sum^{}pi*\left(1-pi\right)\;for\;i=1,\ldots n$$

A decision tree is easy to understand. However, if data contain various features it might cause problems that are called overfitting. Therefore, it is crucial to know when to stop growing trees. Two methods are typical for restricting the model from overfitting: pre-pruning, which stops growing early, but it is hard to choose a stopping point; and post-pruning, which is a cross-validation used to check whether expanding the tree will make improvements or lead to overfitting [42,43]. DT structure consists of a root node, splitting, decision node, terminal node, sub-tree, and parent node [4]. There are two main phases of the DT induction process: the growth phase and the pruning phase. The growth phase involves a recursive partitioning of the training data resulting in a DT where decision trees have a natural “if”, “then”, “else” construction that makes it fit easily into a programmatic structure [44]. 

### 3.6. Random and Rotation Forest

Random forest is an ensemble learning model that can be used for both regression and classification. Indeed, a random forest consists of many decision trees. Therefore, in some cases, it is more logical to use random forest rather than a decision tree. 

The rotation forest algorithm consists of generating a classifier that is based on the extraction of attributes. The attribute set is randomly grouped into K different subsets. It aims to create accurate and significant classifiers [45].

In the decision tree, feature selection is the main problem, and there are different approaches for that. Furthermore, random forest searches the best feature among a random subset of features, instead of searching for the most prominent feature while splitting nodes. It is possible to make it even more arbitrary by using random thresholds for each feature rather than seeking the best one [46]. 

Some built-in function parameters could make the model faster or more accurate. Max features, n estimators, and min sample leaf are used for increasing the power of prediction. N jobs and random state are generally used for making models faster. In this study, n estimators, which determines the number of trees to grow, and random state parameters are used to increase the accuracy and speed of the model. In Table 2, parameters that are used in the dataset are represented and explained.

The dataset contains 32 parameters. All parameters can be useful to classify cancer; if these parameters have relatively large values, it can be a sign of malignant tissue. The first parameter is ID, and it is a number that is used for identification [30]. The second parameter is the diagnosis of membranes, of which there are two diagnoses for tissue: malignant and benign. For different cancer types, it is necessary to determine the correct diagnosis of tissue in case both membranes have different treatments. After these two, estimated means, standard errors, and radius means indicate a range between the center and point on the perimeter. Radius se shows the estimated standard error. Radius worst has the highest value of the center for the estimated range. It is essential to know the distance between the center and the point because surgery depends on the size. There is no chance to do surgery with big tumors. Texture mean represents the standard deviation of the gray-scale values. Texture se represents the standard error of the calculated standard deviation for gray-scale values. The highest mean value of standard deviation for gray-scale values is shown as texture worst. Gray-scale is commonly used to find the tumor location, and the standard deviation is essential to find the variation of the data and to explain how to spread out the numbers. Perimeter mean represents the mean value for the core tumor, while standard error of the mean represents the core tumor described as perimeter se. The highest value of the core tumor is written on the perimeter worst column. Area mean, area se, and area worst point at similar values related to the mean of the cancer cell areas, as described before. Smoothness mean is the mean for regional variations in radius range, smoothness se represents standard error of the mean of local variations in radius length, and the largest mean value is shown as smoothness worst. Compactness mean is a mean value of estimation of the perimeter and area, compactness se is used for standard error of compactness mean, and the highest mean value of the calculation is named compactness worst. Concavity mean shows the severity of concave portions of the shape, and concave points mean is the number of concave portions of the contour. Concavity se stands for the standard error of concave portions, while concave points se stands for the standard error of the concave portions of the shape. Concavity worst and concave points worst stand for the highest mean value. Fractal dimension mean is the calculated mean value for coastline approximation, standard error of the coastline approximation is shown as fractal dimension se, and the highest mean value is fractal dimension worst [17,18,30].

R, Matlab, Stata, SAS, WEKA, and Python are popular tools for data analysis and visualization. In the literature, WEKA is also a favorite tool in data science. In recent years, R programming language, Minitab, and Python have shown to one step ahead of their competitors with their packages and libraries. On the other hand, Matlab created its tool for data science to operate with data and features efficiently.

In this study, bestglm, car, corrplot, gpplots, leaps, ROCR, and MASS libraries in R as well as NumPy, Pandas, Matplotlib, Seaborn, Plotly, and Scikit-learn libraries in Python are used for data visualization and implementation of machine learning algorithms. As the first step of this study, the dataset was imported to R, Minitab, and Python as a data frame and examined further. After observing data, two unnecessary columns were extracted from the data set. These columns were removed from the core data, and this process can be called data cleaning. Then, the label column was transferred to another data frame to make steps easier while plotting charts. The label column shows whether the tumor was benign or malignant. 

**Figure 4:**
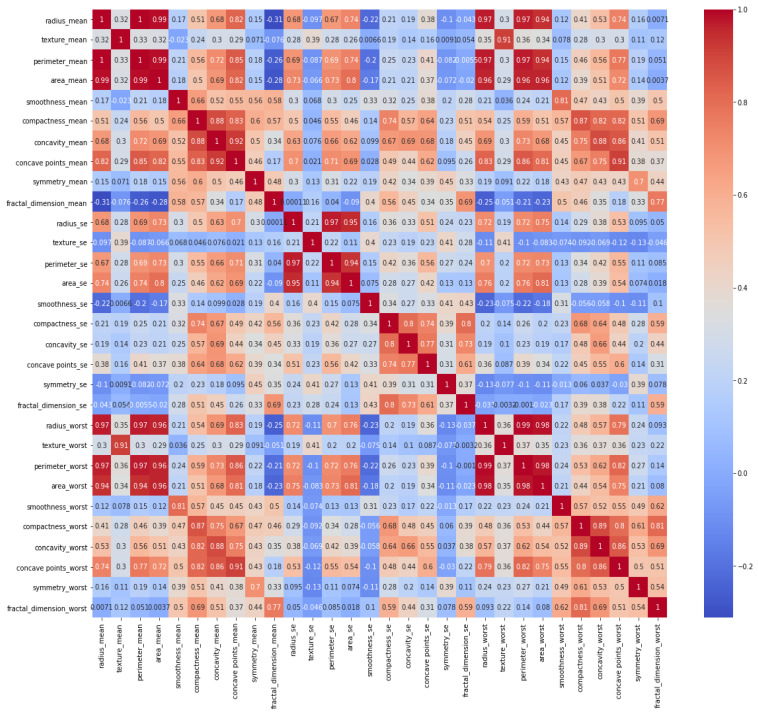
Heat map representative of independent variables.

It is necessary to identify whether data are balanced or unbalanced. It can be observed that the dataset was not smoothly balanced, and the number of benign tumors was almost twice that of malignant tumors. In the next step, a heat map was constructed to indicate a correlation between all features, and its graph is given in Figure 4.

In the dataset, some of the features had higher numerical integer values, yet some of them, on the other hand, had much lower numerical values. Plotting charts and relations among features and unbalanced numerical values would not give adequate outcomes. Consequently, numerical values are normalized. The normalization equation of numerical values can be estimated as

(6) $$i^{*}=\frac{i-\mu }{\sigma }$$

i=each unique value in the data

**Figure 5:**
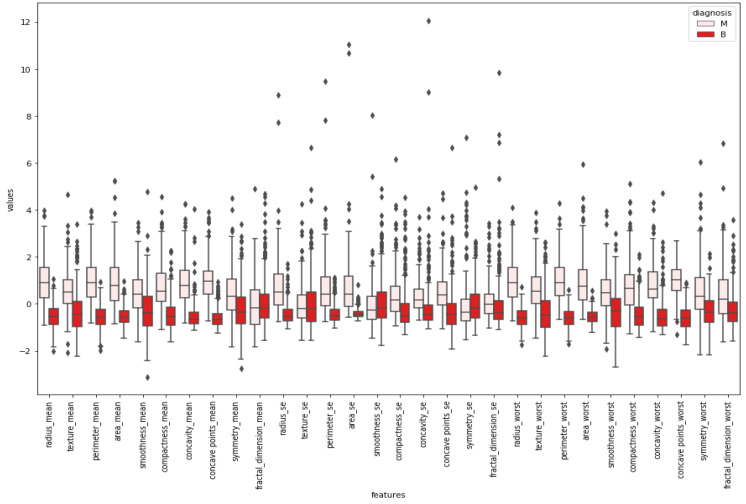
Boxplots.

After normalizing the numerical values of features, a box plot was created for cleaning the data. Figure 5 indicates a box plot of features. According to it, some features were excluded since there were too many outliers. This means sufficiency was lacking to use these features while classifying by looking at the boxplot. 

**Figure 6:**
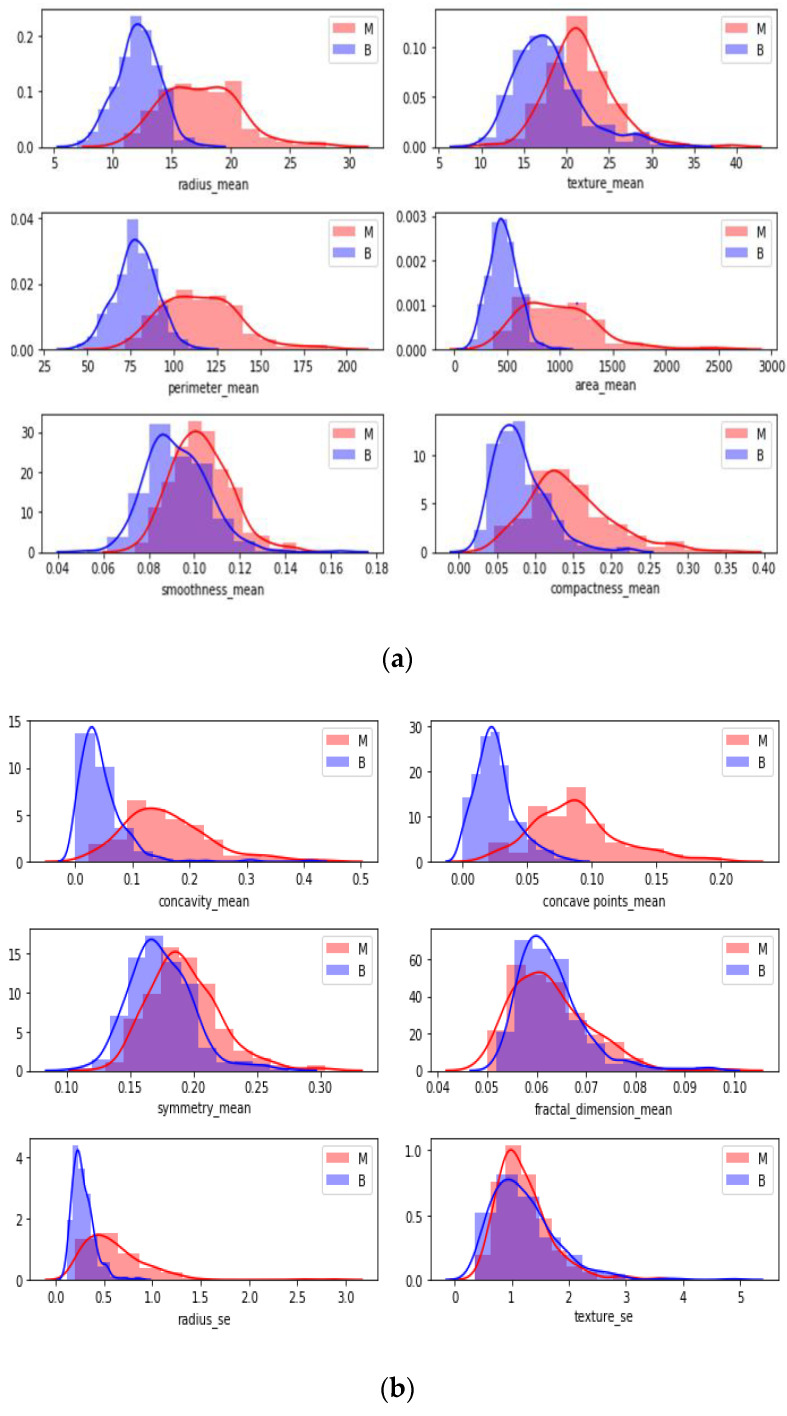

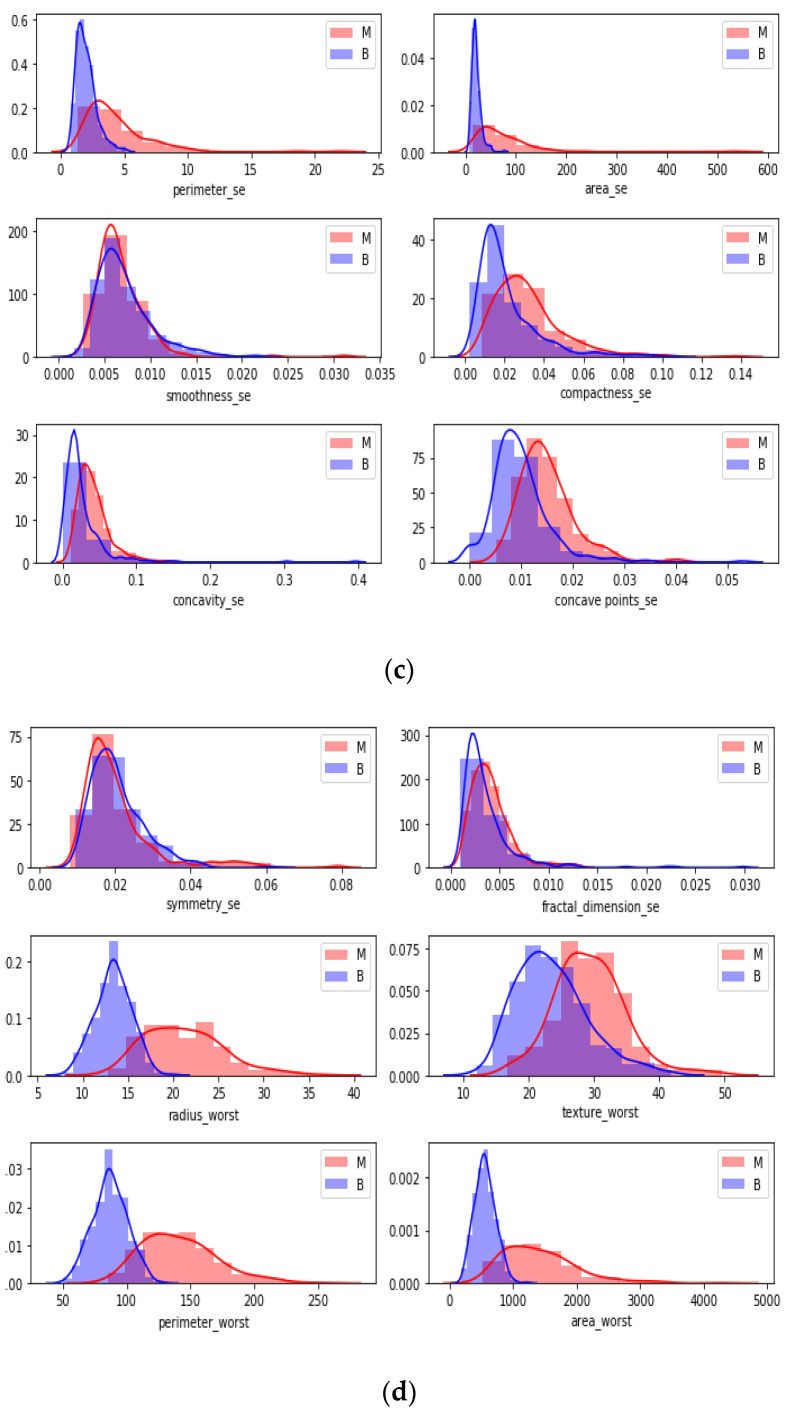
Pairwise plots of Breast Cancer Dataset attributes.

Swarm plot provides more visualization rather than a box plot for classifying. According to the first swarm plot, redundant features were excluded, and the final swarm plot was generated. Before machine learning techniques, data were grouped under three main categories, which were positively correlated, negatively correlated, and uncorrelated, then drawn as scatter plots in Figure 6.

## 4. Results

Supervised learning method includes algorithms for identified data by understanding the given data and making predictions for the future. Classification and regression are two different categories under this approach. Classification is a technique for determining the label of the data and used for discrete responses, unlike the regression technique. In the classification process, the first step is to read the given data. Different classification algorithms are usually preferred for machine learning applications. In this study, logistic regression, k-nearest neighbor, support vector machine, random forest, decision tree, and naïve Bayes classification algorithms were created, and accuracy scores for each of them were obtained. Each algorithm was applied to three different datasets that included various features. The first dataset covered all independent features, the second dataset included highly correlated features, and the last dataset included low correlated features. Three different datasets were used separately for each machine learning technique, and accuracy results were obtained to make comparisons.

Logistic regression is one of the most common algorithms to solve classification problems. It measures the relationship between a categorical dependent variable and independent variables by using a sigmoid function. Before the application, the dataset was divided into two groups, a training set and testing set, for logistic regression. Eighty percent of the data was preferred for the training set, and the rest of it was used for testing to get accurate results from the classification algorithm. The logistic regression algorithm was applied with an optimum cut off value/threshold by using different libraries, and accuracy results were obtained as 98.06%, 95.61%, and 93.85%.

The second applied model was KNN. Three different datasets were used with 10, 12, and 10 neighbors sequentially. The random state was defined as 42 for the k-nearest neighbor algorithm as applied before in logistic regression. According to each application, accuracy scores were obtained as 96.49%, 95.32%, and 94.69% for each dataset. 

SVM technique was applied to get better accuracy scores. However, before application of the algorithm, a random state was defined as 1, not 42, since it gave better results. SVM accuracy results were obtained as 96.49%, 96.49%, and 93.85%.

Naïve Bayes method gave the worst accuracy result compared to the other methods. Accuracy results for each dataset were obtained as 94.73%, 92.98%, and 93.85%. 

Decision tree algorithm belongs to the family of supervised learning algorithms. The working principle of it is based on random selections. Positions of the features are selected randomly in the decision tree algorithm. Therefore, when the function runs several times, it is possible to get different accuracy results. In this study, with decision tree function, the best results were received as 95.61%, 93.85, and 92.10%.

Random forest algorithm has the same working principle as a decision tree. In the random forest, there are n number of decision trees. In this application, n was defined as 50, and accuracy results were obtained as 95.61%, 94.73%, and 92.98% for each dataset.

Rotation forest algorithm consists of generating a classifier based on the extraction of attributes. The attribute set is randomly grouped into K different subsets. In this application, the rotation forest algorithm gave competitive results. Accuracy results were obtained as 97.4%, 95.89%, and 92.99% for each dataset.

Among women, 62.7% had a benign tumor type, while 37.3% had a malignant tumor type in the given dataset. This distribution shows that the data were unbalanced, with benign tumors being more frequently stored. The heat map illustrates the correlation between each feature one by one. It contains 900 (30 feature x 30 feature) relationships to indicate the relationship within all features. Darker blue colors represent that there was a clear and positive correlation between those features, while lighter blue colors show a negative correlation, and uncorrelated for benign breast mass. Similarly, darker red colors represent that there was a clear and positive relationship between those features, while lighter red colors show a negative correlation and uncorrelated for malignant breast mass. For instance, radius mean and perimeter means had a strong and positive correlation with a 1.0 coefficient value. 

Boxplots were created to give insight into the basic statistics of the data and outliers. Tumor types were divided into their labels, and boxplots were constructed for each feature. According to the boxplot results, features that could classify tumor types better were selected for further applications. Graphical demonstrations in Figure 5 and Figure 6 propose the benefit of differentiating between the shapes of the normal distribution (e.g., smoothness mean) and other distributions. All of the reviewed boxplot variations used the median, and variations rarely presented summary statistics around the mean or a value close to it.

In Figure 6, each attribute distribution indicated concerning tumor types. It describes the distribution of attributes. Firstly Figure 6a,b show the attribute mean and standard error distribution, while Figure 6c,d indicate a standard error and worst value of attributes. For instance, the shape of radius mean attribute distribution for a benign tumor is symmetric, while the area se attribute distribution for a malignant tumor is right-skewed. Attribute data values largely varied for some of the attributes.

**Figure 7:**
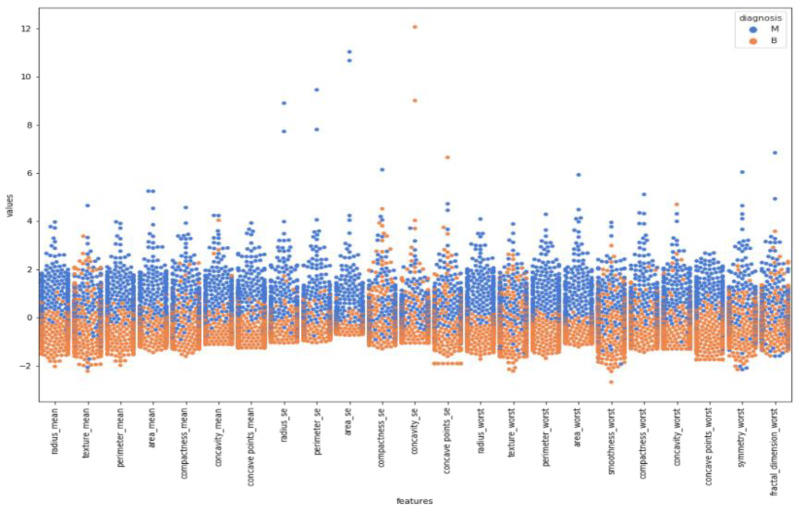
Swarm plot after the first elimination.

In Figure 7, a detailed correlation was investigated by visualizing each individual in the data by using a swarm plot. Features were divided into two groups: uncorrelated and correlated. Uncorrelated and correlated features were two groups that showed high and low correlation features throughout the dataset. The plot indicates that the mean of data distribution was between −2 and +2. 

**Figure 8:**
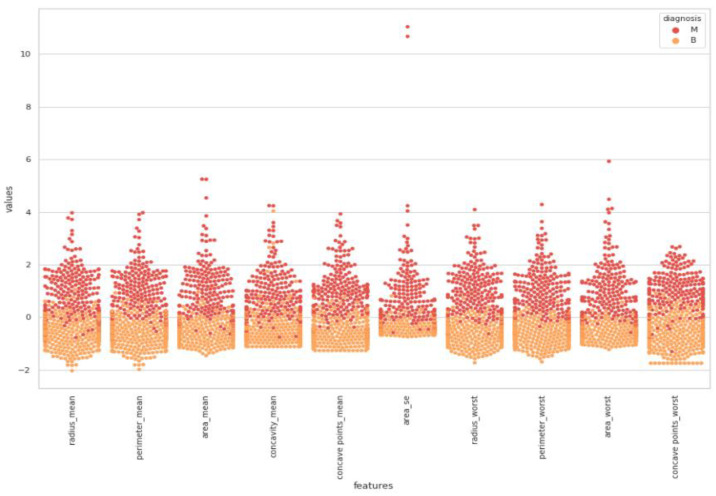
Final swarm plot after second elimination.

In Figure 8, correlated features can be observed with dropping uncorrelated features. It was observed that a comparatively denser and higher correlation existed. Nodes stayed close to each other.

**Figure 9:**
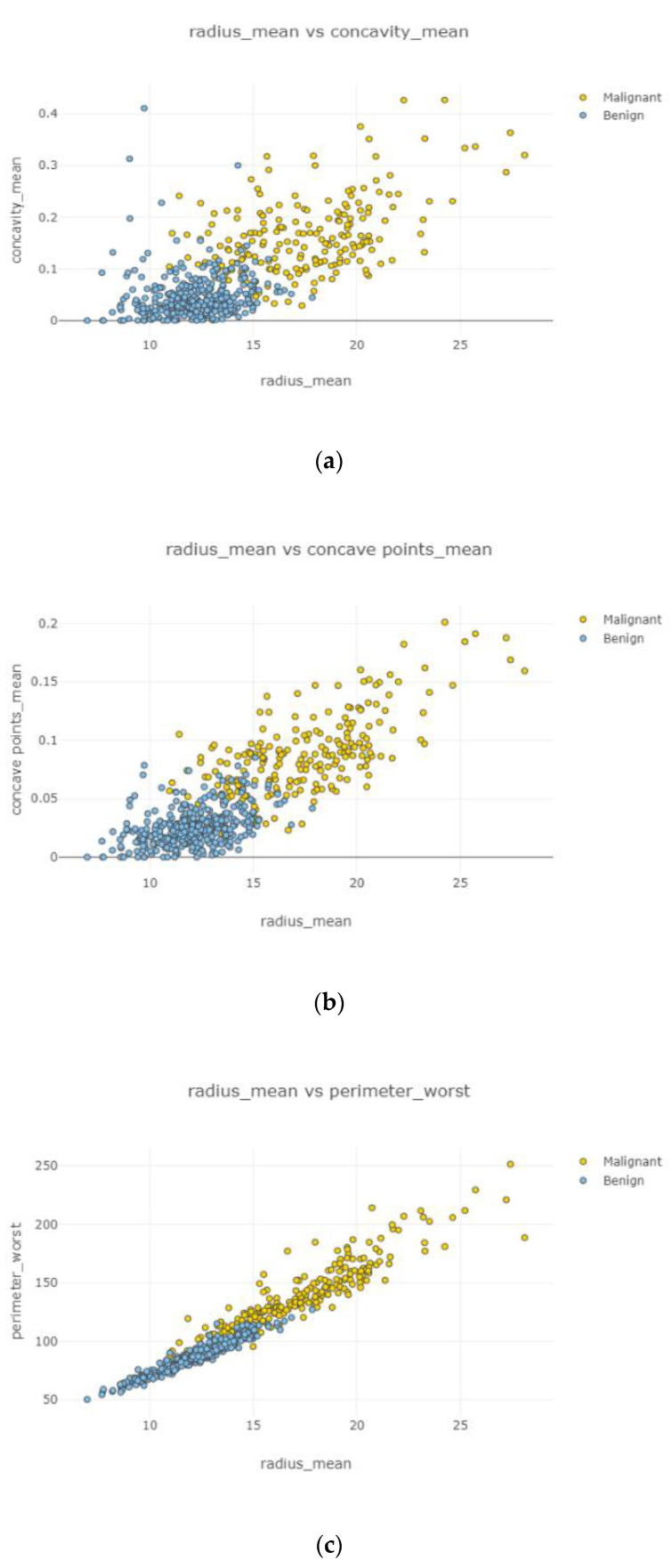

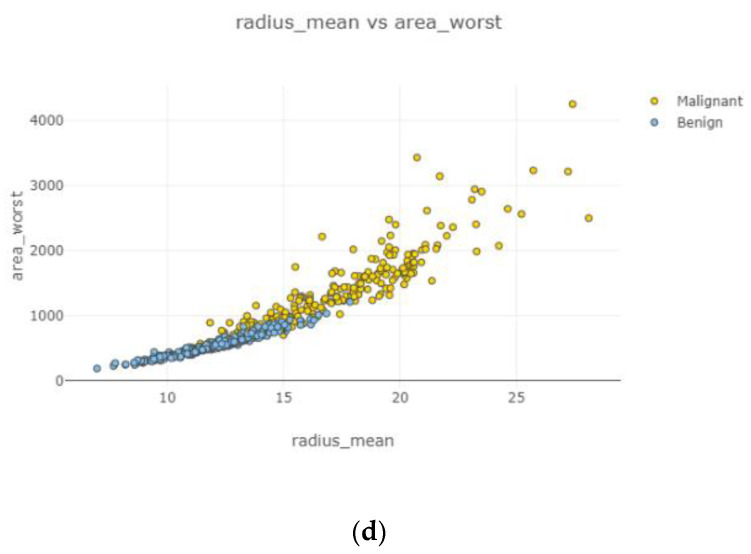
Positively correlated scatter plots 1–4.

In Figure 9, the correlation between features is clear, accurate, and high. Correlated features can be seen with eliminating uncorrelated features. Figure 9c shows the highest correlation between variables, while Figure 9a displays the lowest relationship among two variables. Figure 9d displays a relatively strong correlation rather than Figure 9b. Observations became increasingly correlated as nodes became closer. 

**Figure 10:**
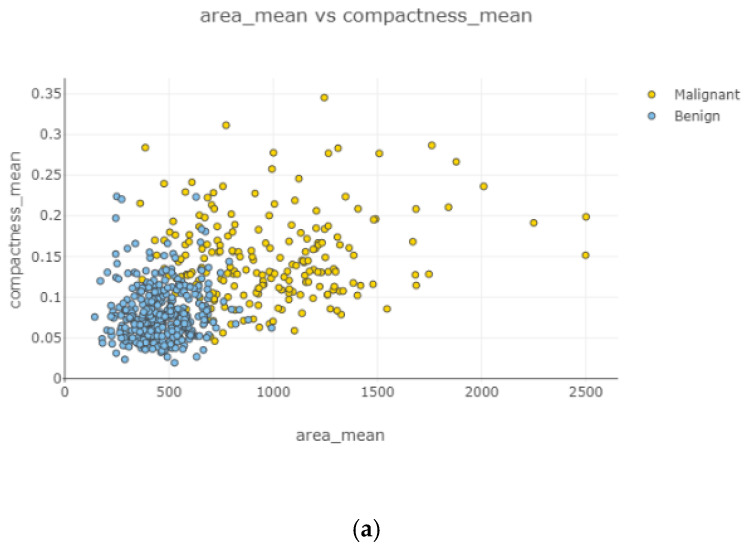

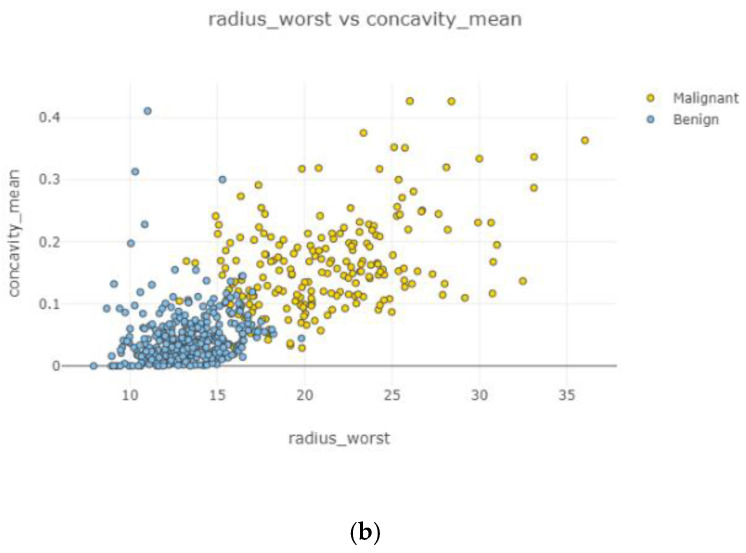
Positively correlated scatter plots 5–6.

In Figure 10, the correlation between features is moderate and positive. Concavity mean and radius worst (Figure 10b) had the highest correlation coefficient severity, while compactness mean and the area mean (Figure 10a) had the lowest correlation coefficient magnitude.

**Figure 11:**
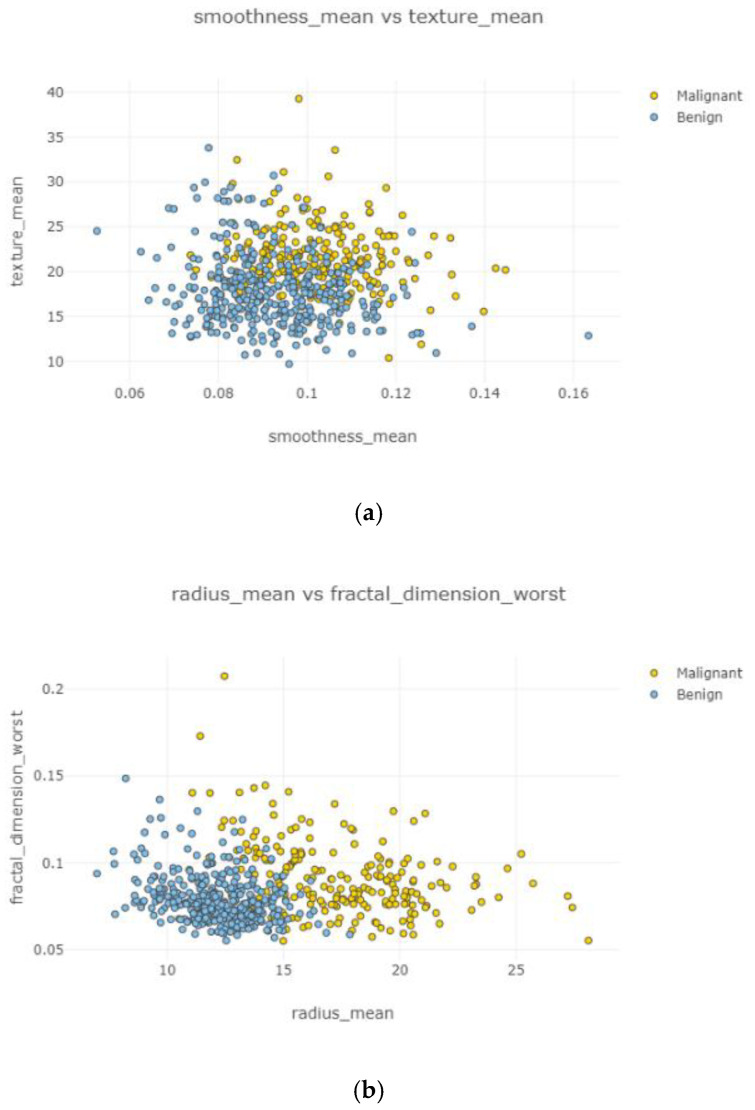

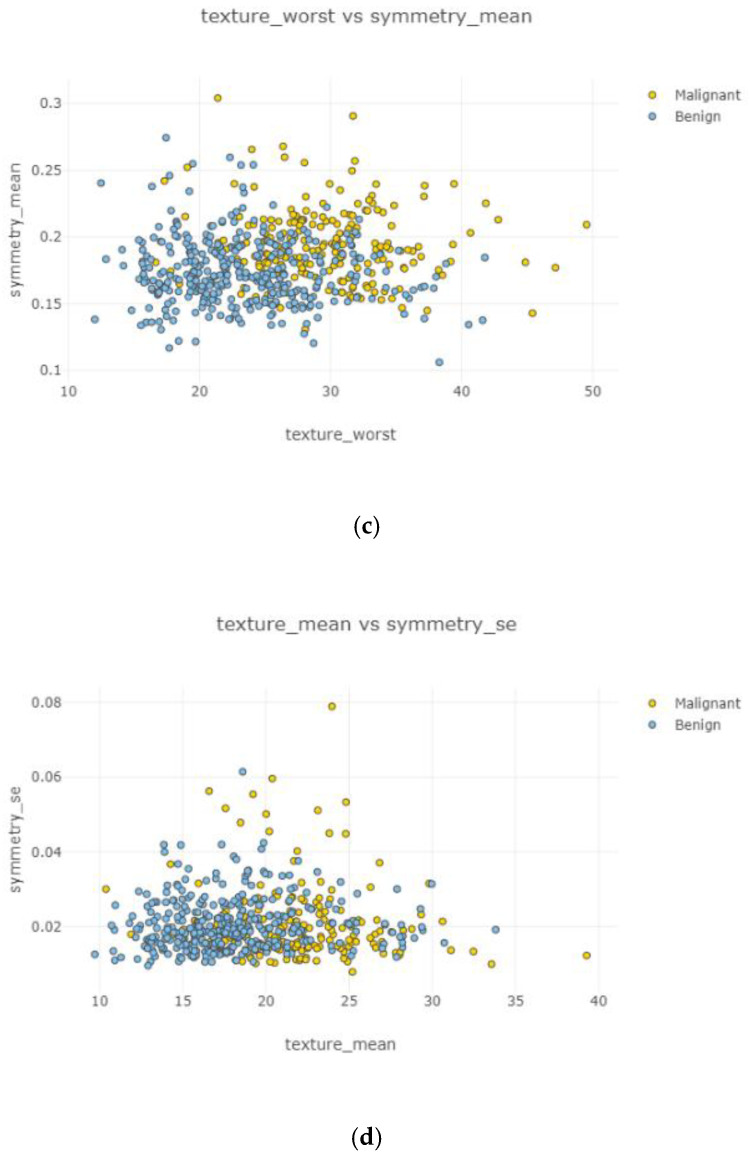
Uncorrelated scatter plots 1–4.

In Figure 11a–d, the correlation between features is weak and irregular. It means there was no correlation between features and did not affect each other.

**Figure 12:**
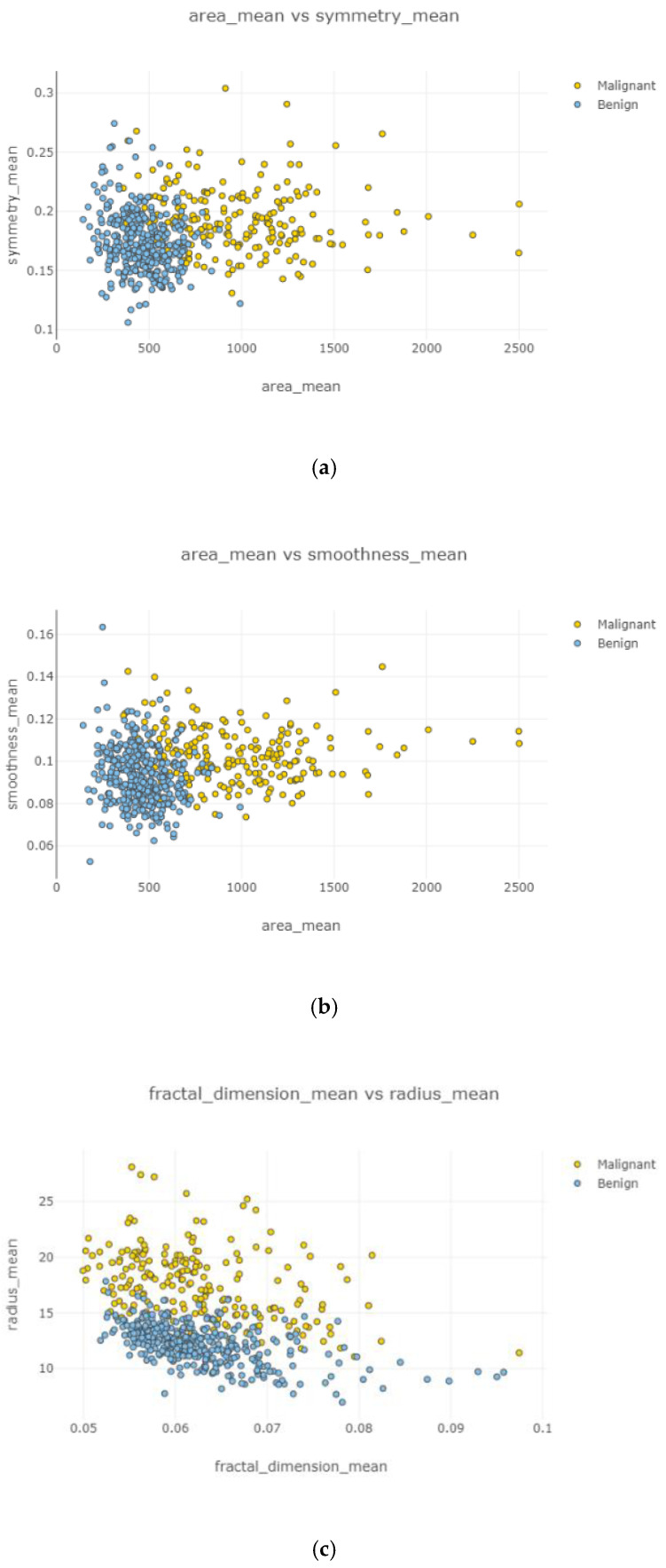

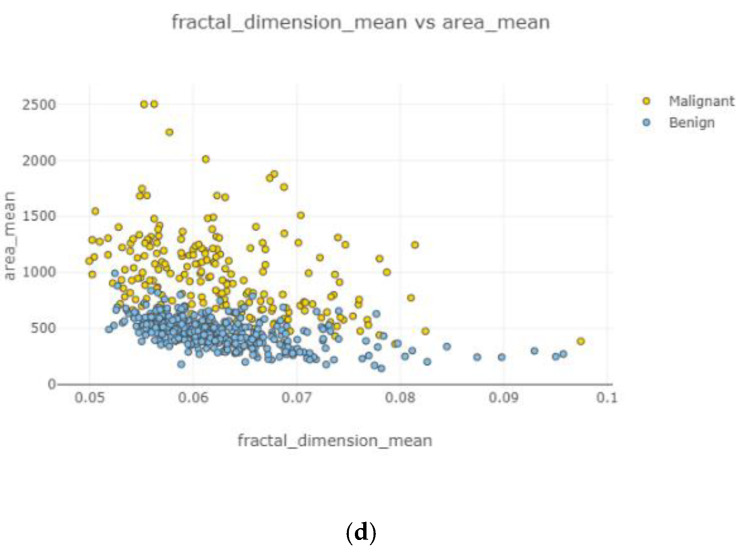
Negatively correlated scatter plots 1–4.

**Figure 13:**
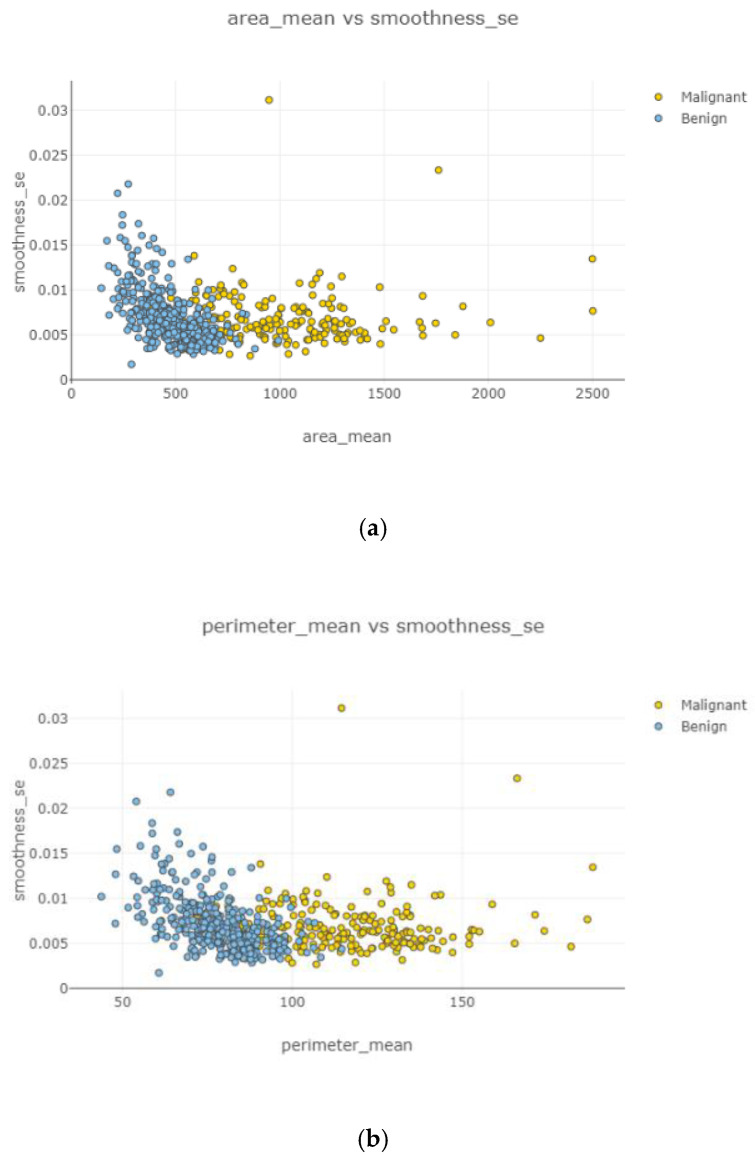
Negatively correlated scatter plots 5–6.

In Figure 12a–d and Figure 13a,b, correlation between features is weak, negative, and high. Correlated features can be seen with eliminating uncorrelated features. Radius mean and fractal dimension mean had the weakest (Figure 12b) correlation value, and smoothness standard error and perimeter mean (Figure 12d) had the highest correlation intensity.

**Figure 14:**
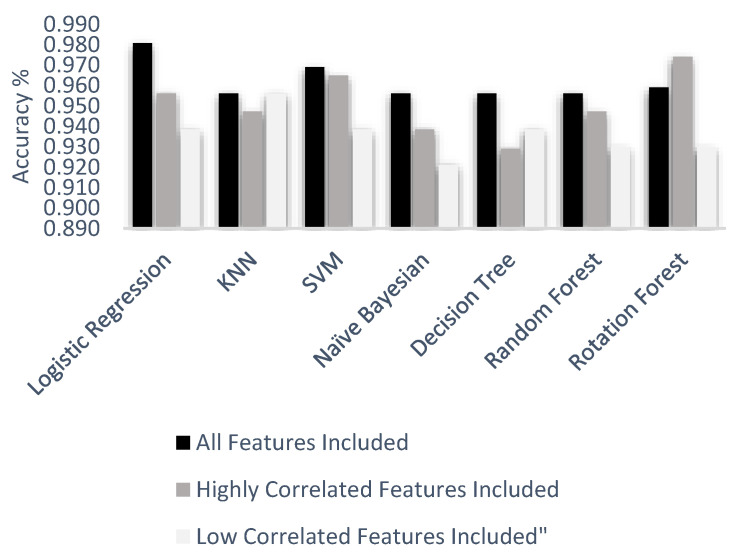
Applied machine learning techniques with accuracy results.

After the data cleaning process, positively correlated, uncorrelated, and negatively correlated groups were created and represented in Figure 8, Figure 9, Figure 10, Figure 11 and Figure 12. Under the positively correlated category, features of radius mean, concavity mean, concave points mean, perimeter worst, area worst, and compactness worst were grouped, and their graphs were plotted. Under the negatively correlated category, smoothness mean, texture mean, radius mean, fractal dimension mean, texture worst, symmetry se, symmetry mean, and the area mean were also grouped, and their graphs were represented in Figure 13a,b. Breast cancer diagnosis using logistic regression had a 98.60% accuracy level for the malign tumor type and 97.17% accuracy level for the benign tumor type. The average accuracy level was 98.07%. Figure 14 indicates a comparison of machine learning techniques with accuracy results.

In Figure 14, all accuracy result values under three feature groups and six machine learning algorithms are given. According to the experiment result, the worst scenario was a decision tree algorithm with low correlated features. Nevertheless, logistic regression with all features included provided the best accuracy result, compared to all other scenarios, with 98.1%. All test accuracy results of all features were 98.1%, 96.9%, 95.9%, 95.6%, 95.6%, and 95.6% from best to worst respectively. Highly correlated test accuracy results were 97.4%, 96.5%, 95.6%, 94.7%, 94.7%, 93.8%, and 93.0% from best to worst respectively. Low correlated test accuracy results were 95.6%, 93.9%, 93.9%, 93.9%, 93.0%, 93.0%, and 92.1% from best to worst respectively.

## 5. Discussion

Data science is a multidisciplinary field that uses scientific methods, processes, algorithms, and systems to extract knowledge and insights from structured and unstructured data. Statistics, data mining, data visualization, machine learning, deep learning, and artificial intelligence are the main subtopics of data science. Even though data science was born in the 1990s, the importance of this field is realized nowadays. It is mentioned in different studies that the amount of data in the world is increasing rapidly, and the unstructured data type still accounts for more than half of the total amount of data. Therefore, data science has become an essential issue in any field to make data understandable. Healthcare is one of the necessary environments for data science applications since big data is a part of it. The volume of collected data in healthcare is enormous, yet it is proven that 80% of the gathered data is unorganized. The total number of studies among data science applications in healthcare has increased significantly [45].

The objectives of this study were to analyze the Wisconsin breast cancer dataset by visualizing it and classify tumor types, whether benign or malignant, by using machine learning algorithms. As a first step, the dataset was prepared for visualization by removing non-numerical values and normalizing each numeric value. Then, heat map, boxplot, swarm plot, and scatterplots were created by using R studio, Minitab, and Python. Visualizing the data helped to understand the correlation between each feature and brought out unnecessary features that were not essential to use while making predictions [47]. After visualization was completed, three different datasets were generated. The first dataset covered all features, the second one included highly correlated features, and the last one included features with a low correlation. Machine learning algorithms, which were logistic regression, k-nearest neighbor, support vector machine, naïve Bayes, decision tree, random forest, and rotation forest, were applied for classification of the tumor type. Accuracy results were obtained for the three different datasets and given in a table as a result [48]. Logistic regression gave better accuracy results rather than the other methods. The main advantage of LR is that it is very efficient to train. In addition, the LR model is useful and gives more accurate results in complex algorithms.

Attributions to the cause of death in those with breast cancer may depend on numerous reasons related to the specifications of the patient. Any specific cause can reduce the risk of death as a result of breast cancer. Nonetheless, these results underline the significance of early diagnoses faced by both active and former patients with a history of breast cancer. Our study reinforces the importance of early diagnosis with high accuracy in women with breast cancer.

In this paper, six distinct machine learning techniques were investigated for breast cancer diagnosis. A competitive performance was demonstrated when dealing with imbalanced data (98.1% accuracy). However, it is essential that before running the algorithm, the dataset must be pre-processed, as it does not deal with missing values, and it has a better performance when learning from a dataset with discretized nominal values. 

## Figures and Tables

**Table 1:** Recent Related Studies.

Objective	Study	Approach and Methods Used
**Clarification of data science and applications**	Dhar [14]	Literature reviews
	Aruna et al. [22]	Naïve Bayes, support vector machine, decision tree
	Chaurasia et al. [23]	Naïve Bayes, SVM, neural networks, decision tree
	Asri et al. [24]	SVM, decision tree (c4.5), naïve Bayes, k-nearest neighbors
	Delen et al. [25]	Naïve Bayes, neural network, c4.5 decision tree
	Qu et al. [26]	Naïve Bayes, decision tree, random tree
	Sriniva et al. [27]	One dependency augmented naïve Bayes, naïve Bayes
**Prediction of breast cancer by using data mining methods**	Bernal et al. [28]	Logistic regression, neural networks, decision tree, nearest neighbors
	Wang et al. [29]	Support vector machine (SVM), artificial neural network (ANN), naïve Bayes classifier, adaboost tree
	Williams et al. [31]	Naïve Bayes and the J48 decision trees
	Nithya et al. [32]	Naïve Bayes, support vector machine-sequential minimal optimization, decision tree, multilayer perceptron
	Oyewola et al. [33]	Logistic regression, linear discriminant analysis, quadratic discriminant analysis, random forest and support vector machine
	Agarap [34]	Gru- SVMS, linear regression, multilayer perceptron, nearest neighbor, softmax regression and support vector machine
	Westerdijk [35]	Logistic regression, random forest, support vector machine, neural network, and ensemble models
	Vard et al. [36]	Particle swarm optimization (PSO), support vector machine (SVMs), decision tree and multilayer perceptron neural network
	Kourou et al. [37]	Artificial neural networks (ANNs), Bayesian networks (BNs), support vector machines (SVMs) and decision trees (DTs)
	Pratiwi [38]	Extreme learning machine methods
	Shukla et al. [39]	Self-organizing map (SOM) and density-based spatial clustering of applications with noise (DBscan), multilayer perceptron (MLP), SEER program

**Table 2:** Wisconsin breast cancer dataset.

1	ID	9	Symmetry Mean	17	Smoothness Se	25	Perimeter Worst
2	diagnosis	10	concavity mean	18	compactness se	26	area worst
3	radius mean	11	concave points mean	19	concavity se	27	smoothness worst
4	texture mean	12	fractal dimension mean	20	concave points se	28	compactness worst
5	perimeter mean	13	radius se	21	symmetry se	29	concavity worst
6	area mean	14	texture se	22	fractal dimension se	30	concave points worst
7	smoothness mean	15	perimeter se	23	radius worst	31	symmetry worst
8	compactness mean	16	area se	24	texture worst	32	fractal dimension worst
